# Anaesthetic Management and Peripartum Outcomes for Parturients With Valvular Heart Disease in a Tertiary Care Hospital of Pakistan

**DOI:** 10.7759/cureus.37666

**Published:** 2023-04-17

**Authors:** Samina Ismail, Sana Urooj

**Affiliations:** 1 Department of Anaesthesiology, Aga Khan University Hospital, Karachi, PAK; 2 Department of Anaesthesiology/Pain Management/Surgical ICU, Dr. Ruth K. M. Pfau Civil Hospital Karachi, Dow University of Health Sciences, Karachi, PAK

**Keywords:** maternal and neonatal complications, anaesthetic technique, mode of delivery, rheumatic heart disease, anaesthetic management, low-middle-income countries, valvular heart disease

## Abstract

Introduction

Parturients with valvular heart disease are at increased risk of maternal cardiac and neonatal complications. We aim to observe maternal cardiac complications in relation to the type of anaesthesia and mode of delivery as our primary objective and neonatal complications as the secondary outcomes.

Methods

We retrospectively reviewed all parturients with valvular heart disease undergoing delivery over a five-year period at the Aga Khan University Hospital, Karachi, Pakistan. to identify maternal cardiac and neonatal complications occurring during the peripartum period.

Results

Of 83 patients with valvular heart disease, 79.5% had rheumatic heart disease. Caesarian section (CS) was performed in 79.5% of patients and regional anaesthesia (RA) was given to 62.1%. Patients with cardiac risk index > 2 were delivered by CS and 64.5% received RA. One maternal and three neonatal deaths were reported with a complication event rate of 9.64% in parturients and 40.9% in neonates. Incidence of maternal cardiac events was one in 17 (5.8%) for vaginal deliveries versus seven in 66 (10.6 %) for CS. Maternal events for CS under RA was 5/66 (7.5 %) vs 2/66 (3%) under general anaesthesia. The incidence of peripartum maternal cardiac events when stratified by severity of cardiac disease was similar to a previously derived cardiac risk index for pregnant women with cardiac disease with no statistical difference in the adverse events rate from the estimated rates (p-value= 0.42).

Conclusion

Elective CS with RA was a common approach for high-risk parturients; however, the benefits cannot be ascertained. Despite low maternal and neonatal mortality, significant maternal cardiac and neonatal complications were observed.

## Introduction

Pre-existing cardiac disease contributes largely to maternal mortality rate worldwide, particularly in low-middle-income countries (LMIC) [1]. Rheumatic heart disease (RHD) is prevalent in LMIC, as 88-90% of the cardiac disease among parturients from these countries is due to RHD [1]. The World Health Organization and the World Heart Federation are striving to reduce mortality from cardiac disease by 25% [2].

Valvular heart disease due to RHD has declined in developed nations but is still affecting women from developing countries [3]. Countries like India, Indonesia, Pakistan, the Democratic Republic of Congo, and China have a high prevalence of RHD accounting for 73% of cases diagnosed worldwide [4]. Child-bearing women are affected the most in countries where RHD is endemic with an estimation of age-standardization prevalence greater than 1% [2].

Pregnancy is associated with significant cardiovascular physiological changes, which are poorly tolerated in parturients with severe valvular heart disease. This leads to deterioration in their clinical condition resulting in maternal and neonatal complications. Neonates born to these mothers may suffer from unfavourable outcomes like premature birth, low birth weight, and even death [4]. Sui et al. proposed a risk index score for cardiac disease in pregnancy (CARPREG) to predict pregnancy outcomes [5], which was further validated by Khairy et al. in a population of parturients predominated with congenital valvular heart disease [6]. There is limited data to predict pregnancy-related outcomes in parturients suffering from RHD [7]. Previous studies of parturients with cardiac diseases have shown a high rate of caesarean section (CS) [5], but lack data on the pregnancy-related outcomes associated with anesthetic management in pregnant women with RHD. 

Even though rheumatic fever is prevalent in developing countries like Pakistan [8,9], and is the leading cause of valvular heart disease in parturients, no data is available related to anaesthetic management and its effect on maternal and fetal outcomes. Therefore, the aim of this study is to examine the association of anaesthetic management and mode of delivery with pregnancy-related outcomes in parturients suffering from valvular heart disease.

## Materials and methods

After the Institutional Ethical Review Committee exemption (ERC no: 3146-Ane-ERC-15), this retrospective study was conducted at the Aga Khan University Hospital, Karachi, Pakistan. Informed consent from the patients was not required as patients were not directly involved, and the hospital computerized database was used for data collection. The word “cardiac disease” was searched from the hospital computer database of obstetric patients who delivered within five calendar years from January 2015 to 2019. The medical records of these patients were then retrieved for data collection. The identity of patients was not disclosed, and their hospital identification number was kept confidential with the investigators. In our hospital setting, pregnant women with either established heart disease or diagnosed during pregnancy are referred to cardiologists to undergo standardized cardiac evaluation and optimization during pregnancy. All newborns born to high-risk patients are examined by a neonatologist and documented in the medical record of neonates.

The retrieved medical records were then independently reviewed by the two investigators to narrow down the search for only those patients with either acquired or congenital valvular heart disease with pregnancies of more than 28 weeks of gestation. The medical record numbers of neonates born to these mothers were used to retrieve the neonate files.

The data collection form was developed by the investigators, first by piloting data collection on 10 files. Changes were incorporated in the form according to the availability of data. The finalized form was then used for the collection of data by one of the investigators, which included the information on parturient age, current and previous medical and obstetric history, functional status on the first antenatal visit using the New York Heart Association (NYHA) scale, history of previous cardiac events including arrhythmia, heart failure, transient ischemic attack or stroke, hypoxemia with oxygen saturation less than 90% on room air, and previous cardiac surgery or interventions. Additionally, data on the method of delivery (either vaginal route or abdominal route by CS) was recorded, the reason for CS under fetal, obstetric, or maternal cardiac indications, and the technique of anaesthesia as to whether RA or general anaesthesia used was recorded in the data collection form.

A cardiac risk index formulated by Siu and colleagues [5] was used for evaluating each pregnancy. This risk index comprised four predictors for maternal cardiac complications which include the following: (i) NYHA functional class greater than II or hypoxemia, (ii) previous cardiac events or arrhythmia, (iii) reduced left ventricular systolic dysfunction, and (iv) left heart obstruction (mitral stenosis, aortic coarctation, and sub-valvular, valvular, and supravalvular aortic stenosis). One of the investigators, after the review of the medical records, judged retrospectively the presence of each predictor. One point was given to each predictor and the risk index was then calculated as the sum of predictor points with a maximum score of four [5].

The primary outcomes of this study were maternal cardiac events and the secondary outcomes were neonatal events during the peripartum period. Maternal cardiac events included (i) new-onset heart failure, (ii) symptomatic arrhythmia requiring treatment, (iii) stroke or transient ischemic attack of cardiac origin, and (iv) cardiac death. Neonatal events included prematurity, if delivery occurred at less than 37 weeks of gestation, low birth weight (<10th percentile) according to gestational age, intraventricular haemorrhage after birth, transient tachypnoea of the new-born, respiratory distress syndrome, or fetal or neonatal death. Fetal death was recorded if it happened after 20 weeks of gestation and before birth and neonatal death were labelled if it occurred from birth to age 28 days.

Statistical analyses

All statistical analysis was performed using IBM SPSS Statistics for Windows, Version 19.0 (Released 2010; IBM Corp., Armonk, New York, United States). The normality of the quantitative data was checked by the Kolmogorov-Smirnov test. The mean and standard deviation were estimated for quantitative variables. The mode of delivery (vaginal and CS) was stratified, and then all patient characteristics are studied in terms of frequencies and percentages within each mode of delivery and overall. Proportion and percentage were computed for qualitative observation and analyzed by the Chi-square test. To validate the previously used cardiac risk index, actual and predicted cardiac complications were compared by using the Fisher’s exact test and the P-value <0.05 was considered statistically significant.

## Results

During the study period of five years from January 2015 to 2019, there were 17,489 deliveries at our institution and 189 (1.01%) occurred in patients with miscellaneous cardiac diseases. The investigators after reviewing the medical records of the 189 patients, found 83 obstetric patients with valvular heart disease with more than 28 weeks of gestation. None of the patients had repeat pregnancy or twin pregnancy in our record, making a total of 83 cases to be included in our study.

The baseline characteristics of mothers and newborn gestational weeks at delivery based on the method of delivery and the technique of anaesthesia are summarized in Table 1. The majority of the patients belonged to NHYA class 11 (73.5%) and only 6% belonged to NHYA 111 classification. The majority of the patients (79.5%) were born by CS. RA was the preferred technique and was given to 41 (62.1%) patients; among them, 21 (33.3%) were given spinal, nine (13.6 %) received combined spinal‐epidural (CSE), and 11 (16.6%) had epidural as the technique of anaesthesia.

**Table 1:** Baseline maternal characteristics, caesarean section (elective/ emergency), and neonatal gestational age in relation to mode of delivery and anaesthesia technique RA: regional anaesthesia; GA: general anaesthesia; NYHA: New York Heart Association

Variable	Vaginal delivery (n=17), n (%)		Caesarean delivery (n=66), n (%)			Total (n=83), n (%)
	With RA (n=2)	Without RA (n=15)	With RA (n=41)	With GA (n=25)		
Mother's age at delivery (years)						
<20	0 (0%)	0 (0%)	2 (4.88%)		1 (4.00%)	3 (3.61%)
21-35	2 (100%)	15 (100%)	31 (75.6%)		18 (72.0%)	66 (79.5%)
>35	0 (0%)	0 (0%)	8 (19.5%)		6 (24.0%)	14 (16.9%)
Parity before delivery						
0	0 (0%)	1 (6.67%)	9 (22.0%)		7 (28.0%)	17 (20.5%)
1-15	2 (100%)	14 (93.3%)	32 (78.0%)		18 (72.0%)	66 (79.5%)
NYHA Class						
I	1 (50.0%)	5 (33.3%)	7 (17.1%)		4 (16.0%)	17 (20.5%)
II	1 (50.0%)	10 (66.7%)	31 (75.6%)		19 (76.0%)	61 (73.5%)
III	0 (0%)	0 (0%)	3 (7.32%)		2 (8.00%)	5 (6.02%)
Family history						
Yes	1 (50.0%)	5 (33.3%)	18 (43.9%)		7 (28.0%)	31 (37.3%)
No	1 (50.0%)	10 (66.7%)	23 (56.1%)		18 (72.0%)	52 (62.7%)
Kind of caesarean section						
Elective	0 (0%)	0 (0%)	25 (61.0%)		15 (60.0%)	40 (60.6%)
Emergency	0 (0%)	0 (0%)	16 (39.0%)		10 (40.0%)	26 (39.4%)
Obstetric comorbidities						
Yes	1 (50.0%)	3 (20.0%)	12 (29.3%)		3 (12.0%)	19 (22.9%)
No	1 (50.0%)	12 (80.0%)	29 (70.7%)		22 (88.0%)	64 (77.1%)
Non-obstetric comorbidities						
Yes	0 (0%)	0 (0%)	3 (7.32%)		1 (4.00%)	4 (4.82%)
No	2 (100%)	15 (100%)	38 (92.7%)		24 (96.0%)	79 (95.2%)
Newborn's gestational age at delivery						
Preterm (<37 weeks)	0 (0%)	4 (26.7%)	21 (51.2%)		11 (44.0%)	36 (43.4%)
Term (≥ 37 weeks)	2 (100%)	11 (73.3%)	20 (48.8%)		14 (56.0%)	47 (56.6%)

The initial diagnosis of the valve and pathology involved (stenosis or regurgitation), type of disease (congenital or acquired), time of diagnosis (before or after pregnancy) and surgical intervention in form of previous cardiac surgery in accordance with the mode of delivery, and technique of anaesthesia given to parturient is summarized in Table 2. When more than one valvular pathology is present, only the primary one is mentioned. The majority of the pathological lesions were acquired (79.5%) and involved mitral valves (71.1%). Even though the majority (73.5%) had their diagnosis prenatally, only 20.5% had undergone surgical intervention for cardiac valvular pathology.

**Table 2:** Initial diagnosis of the valve and pathology, time of diagnosis, and previous surgical intervention in relation to mode of delivery and anaesthesia technique. RA: regional anaesthesia; GA: general anaesthesia

Variable	Vaginal delivery, n (%)		Caesarean delivery, n (%)			Total (n=83), n (%)	
	With RA (n=2)	Without RA (n=15)	With RA (n=41)	With GA (n=25)			
Valve involved							
Mitral	2 (100%)	13 (86.7%)	27 (65.9%)		17 (68.0%)		59 (71.1%)
Tricuspid	0 (0%)	0 (0%)	2 (4.88%)		0 (0%)		2 (2.41%)
Aortic	0 (0%)	1 (6.67%)	1 (2.44%)		5 (20.0%)		7 (8.43%)
Pulmonary	0 (0%)	0 (0%)	1 (2.44%)		0 (0%)		1 (1.20%)
Mitral + Aortic	0 (0%)	0 (0%)	5 (12.2%)		0 (0%)		5 (6.02%)
Mitral + Tricuspid	0 (0%)	1 (6.67%)	5 (12.2%)		3 (12.0%)		9 (10.8%)
Pathology Involved							
Stenosis	1 (50.0%)	3 (20.0%)	5 (12.2%)		5 (20.0%)		14 (16.9%)
Regurgitation	1 (50.0%)	10 (66.7%)	22 (53.7%)		10 (40.0%)		43 (51.8%)
Both	0 (0%)	2 (13.3%)	14 (34.1%)		10 (40.0%)		26 (31.3%)
Type of disease							
Congenital	0 (0%)	2 (13.3%)	9 (22.0%)		6 (24.0%)		17 (20.5%)
Acquired	2 (100%)	13 (86.7%)	32 (78.0%)		19 (76.0%)		66 (79.5%)
Time of diagnosis							
Before pregnancy	2 (100%)	9 (60.0%)	31 (75.6%)		19 (76.0%)		61 (73.5%)
After pregnancy	0 (0%)	6 (40.0%)	10 (24.4%)		6 (24.0%)		22 (26.5%)
Previous cardiac surgery							
Yes	1 (50.0%)	1 (6.67%)	8 (19.5%)		7 (28.0%)		17 (20.5%)
No	1 (50.0%)	14 (93.3%)	33 (80.5%)		18 (72.0%)		66 (79.5%)

**Figure 1:**
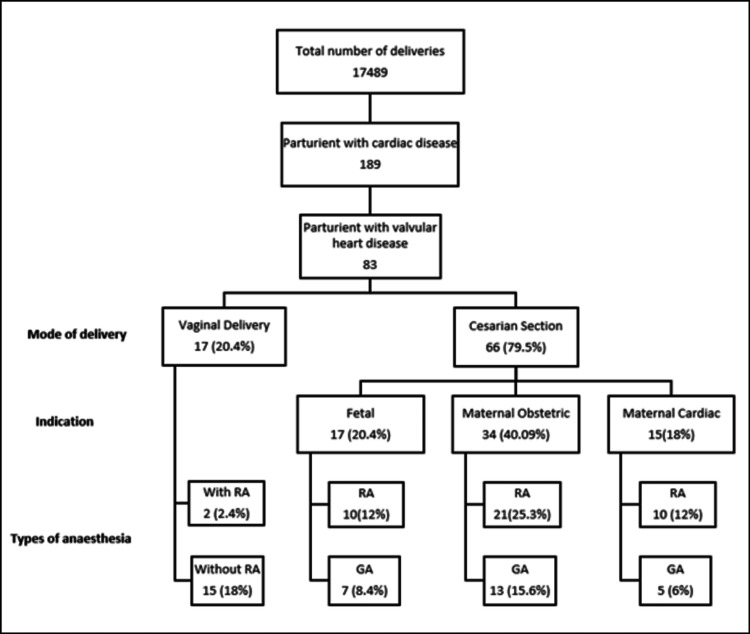
Mode of delivery, indication for caesarean section, and anaesthetic technique GA: general anaesthesia; RA: regional anaesthesia

Anaesthetic technique, mode of delivery, and indication for CS are shown in Figure 1. Vaginal deliveries are categorized into two groups: those with or without the use of regional analgesia. Similarly, CS are subdivided according to the indication and type of anaesthesia for CS. Indications for CS were either fetal, obstetric, or maternal cardiac and if there were more than one indication, only the primary indication for CS was chosen with maternal cardiac reason prioritized. In the majority of the patients (40.9%), the indication of CS was due to maternal obstetric reasons, with maternal cardiac reason seen in only 18% of patients. Further categorization is according to the technique of anaesthesia provided (GA or RA). Parturients in labour requiring CS were listed in the CS group. 

Predictors for maternal cardiac complications, cardiac risk index, adverse events, and maternal place of disposition (post anaesthesia care unit, high dependency unit, intensive care unit, coronary care unit) and neonatal place of disposition (neonatal intensive care unit) in relation to the mode of delivery and anaesthesia technique is summarized in Table 3. Patients having a risk index of 4, had CS as the mode of delivery and RA as the technique of choice. There were five patients with a risk index of 4 and all received RA; one received epidural and four received CSE.

**Table 3:** Predictors of maternal cardiac complication, risk Index, maternal and neonatal events, and disposition in relation to mode of delivery and anaesthesia technique RA: regional anaesthesia; GA: general anaesthesia; RI: risk index; PACU: post anaesthesia care unit; HDU: high dependency unit; ICU: intensive care unit; CCU: coronary care unit; NICU: neonatal intensive care unit; NYHA: New York Heart Association;

Variable	Vaginal delivery, n (%)		Caesarean delivery, n (%)		Total (n=83), n (%)
	(n=17)		(n=66)		
	With RA	Without RA	With RA	With GA	
	(n=2)	(n=15)	(n=41)	(n=25)	
Prior cardiac event/arrhythmias					
Yes	1 (50.0%)	0 (0%)	5 (12.2%)	2 (8.00%)	8 (9.64%)
No	1 (50.0%)	15 (100%)	36 (87.8%)	23 (92.0%)	75 (90.4%)
NYHA Class					
I	1 (50.0%)	5 (33.3%)	7 (17.1%)	4 (16.0%)	17 (20.5%)
II	1 (50.0%)	10 (66.7%)	31 (75.6%)	19 (76.0%)	61 (73.5%)
III	0 (0%)	0 (0%)	3 (7.32%)	2 (8.00%)	5 (6.02%)
Hypoxemia					
Yes	0 (0%)	2 (13.3%)	9 (22.0%)	5 (20.0%)	16 (19.3%)
No	2 (100%)	13 (86.7%)	32 (78.0%)	20 (80.0%)	67 (80.7%)
Reduced ventricular systolic dysfunction					
Yes	0 (0%)	0 (0%)	4 (9.76%)	0 (0%)	4 (4.82%)
No	2 (100%)	15 (100%)	37 (90.2%)	25 (100%)	79 (95.2%)
Left heart obstruction					
Yes	0 (0%)	0 (0%)	5 (12.2%)	1 (4.00%)	6 (7.23%)
No	2 (100%)	15 (100%)	36 (87.8%)	24 (96.0%)	77 (92.8%)
Risk index (number of predictors of maternal complications)					
RI 1	1 (50.0%)	13 (86.7%)	30 (73.2%)	20 (80.0%)	64 (77.1%)
RI 2	1 (50.0%)	2 (13.3%)	6 (14.6%)	2 (8.00%)	11 (13.3%)
RI 3	0 (0%)	0 (0%)	0 (0%)	3 (12.0%)	3 (3.61%)
RI 4	0 (0%)	0 (0%)	5 (12.2%)	0 (0%)	5 (6.02%)
Maternal event					
Yes	0 (0%)	1 (6.67%)	5 (12.2%)	2 (8.00%)	8 (9.64%)
No	2 (100%)	14 (93.3%)	36 (87.8%)	23 (92.0%)	75 (90.4%)
Neonatal events					
Yes	0 (0%)	4 (26.6%)	18 (43.9%)	12 (48%)	34 (40.9%)
Postnatal maternal disposition					
PACU	1 (50.0%)	12 (80.0%)	32 (78.0%)	16 (64.0%)	62 (74.7%)
HDU	1 (50.0%)	2 (13.3%)	3 (7.32%)	3 (12.0%)	9 (10.8%)
ICU	0 (0%)	1 (6.67%)	1 (2.44%)	3 (12.00%)	4 (4.82%)
CCU	0 (0%)	0 (0%)	5 (12.2%)	3(12.00%)	8 (8.43%)
Postnatal neonate disposition					
Well baby nursery	2 (100%)	9 (60.0%)	24 (58.5%)	14 (56.0%)	49 (59.0%)
NICU	0 (0%)	6 (40.0%)	16(41.5%)	11 (44.0%)	33 (40.9 %)

**Figure 2:**
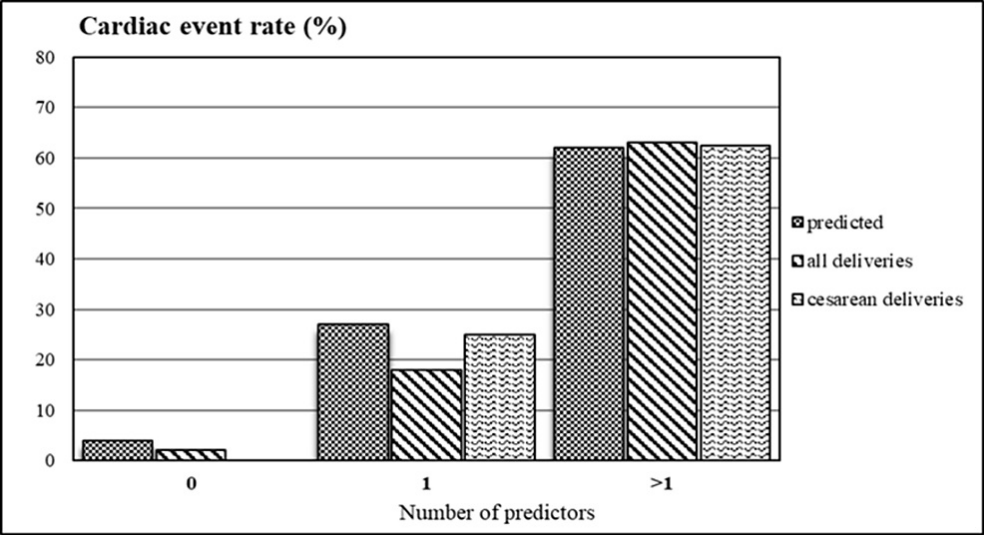
Actual versus predicted maternal cardiac event rates with varying numbers of predictors in all deliveries and caesarean deliveries.

In our study, where the majority of the valvular lesion was acquired secondary to RHD, cardiac adverse events during deliveries including vaginal and CS occurred in 2% (1/64) of patients with cardiac risk index score of 0; 18% (2/11) of patients with a score of 1, and 63% (5/8) of patients with a score of > 1. There was no statistical difference in the adverse events rate from the estimated rates (p-value= 0.42) (Figure 2). When limited to CS, cardiac maternal events occurred in 0% (0/50) of patients having a score of 0;25% (2/8) of patients having a score of 1, and 62.5% (5/8) of patients having a score >1 (Figure 2).

Maternal cardiac events occurred in eight (9.64%) patients, there was one maternal death, six patients suffered congestive cardiac failures, and one had symptomatic arrhythmia. Among patients encountering maternal cardiac events, seven parturients delivered by CS and five received RA (Table 3). One maternal death in our study was of a patient who had moderate stenosis of both mitral and aortic valves; she belonged to NHYA III classification and had left heart obstruction. She received GA as the technique of anaesthesia and the cause of mortality was congestive cardiac failure. All patients encountering maternal events were managed either in ICU or the critical care unit (CCU). Except for one maternal death in the postoperative period, other patients responded to medical management.

Neonatal events occurred in 34 (40.9%) neonates, 32 neonates were born prematurely before 37 weeks of gestation, and two had low birth weight (<10th percentile) according to the gestational age. The majority of these neonates (82%; 28/34) were delivered by CS (Table 3). There were three neonatal deaths. All three were premature, born to mothers encountering maternal cardiac events. All were delivered by CS, two had RA and one was given GA. The neonatal intensive care unit (NICU) admission rate was 40.2%, mostly among neonates born to mothers delivered by CS.

## Discussion

Our five-year retrospective review of maternal cardiac and neonatal complications among parturients with valvular heart disease revealed low mortality of mothers and neonates. We found a high prevalence of RHD compared to congenital heart disease among our patient population. A higher percentage of patients were delivered by CS with RA as a preferred technique of anaesthesia especially in patients with a high cardiac risk index.

The present study demonstrated an overall low maternal and neonatal mortality. The probable reasons are multidisciplinary team involvement with better antenatal optimization and a low number of high-risk patients. Patients in the present study were mainly NYHA class I/II (94.5%), which is very similar to the data presented by Goldszmidt et al. (9i.1%) [10], Hidano et al. (100%) [11], Chumpathong et al (100%) [7], Ilhan Yildirim et al. (92.5%) [12], and Sawhney et al. (80.2) [13].

We found a high prevalence of RHD (79.5%) compared to congenital heart disease among our patient population. The pattern is similar to the data from LMIC, where 88-90% of antenatal heart disease is due to RHD [1]. In the present study, the mitral valve was found to be predominantly affected (71.1%) with regurgitation as the primary pathology in more than 50% of the patient population. The Registry of Pregnancy and Cardiac Disease (ROPAC), which is a large prospective cohort of pregnant women with RHD states that parturients with mitral valve regurgitation can survive pregnancy better than those with mitral stenosis [14]. This could be another reason for low maternal mortality in our patient cohort.

One maternal death in our study subject was in a patient who had moderate stenosis of both mitral and aortic valves, who belonged to NHYA III classification and had left heart obstruction. According to ROPAC, mitral stenosis is an independent predictor of adverse maternal cardiac events and patients with severe mitral stenosis have the highest rate of heart failure (49.1%) [14].

Even though the mortality rate was low in our study, we did observe significant maternal and neonatal events among our study population. Our overall maternal events were low compared to studies on RHD patient population [7,15,16]. However, the rate is higher compared to the studies conducted in populations with CHD [11].

The CS rate of 27.5% and 44% quoted in previous studies are much lower compared to the rate of 79.5% in the current study [7,11]. According to the consensus guidelines, except for high-risk disease, CS should typically be reserved for obstetric indications [16,17]. However previous studies have quoted a rate of 33% of primary CS for cardiac indications, which is higher compared to the general population [18,19]. Our study had the lowest rate for maternal cardiac reasons, as the majority of CS were performed for maternal obstetric reasons. However, observed rates of CS are dependent on the differences in decision-making of obstetricians and cardiologists. 

In the present study, 81% of the parturients with valvular heart disease received anaesthetic care and RA was the technique of choice, especially with a higher risk index. Epidural or CSE was preferred over single-shot spinal in patients with cardiac risk index 4. Our results are comparable to the analysis of Goldszmidt et al. [10], where RA was the technique of anaesthesia and epidural anaesthesia was used in 70% of NHYA class III-IV patients. Epidural anaesthesia is preferred over single-shot anaesthesia due to easily adjustable block levels and cardiovascular stability [7,20]. However, in a study by Yıldırım et al., a high rate of GA has been quoted for high-risk parturients with no difference in the newborn APGAR score and maternal postoperative complications and hospital stay between the RA and GA groups [12]. Even though GA seems to have a higher association with higher maternal and neonatal events, consideration should be given to the clinical condition and urgency of surgery. Gestational age and urgency of birth have been suggested as factors increasing the possibility of GA [10]. In the present study, we observed that a lesser number of patients were given RA for emergency CS compared to elective CS.

Maternal events were more in patients receiving RA; the possible explanation is patients with high-risk index were operated on under RA. Similarly, neonatal events occurred more in the RA group probably because they were born to high-risk mothers operated under RA. Like the previous studies, no agreement exists on the anaesthetic technique for maternal and neonatal outcomes. The optimal technique is the one that is based on a thorough understanding of abnormal cardiovascular physiology and how the anaesthetic technique is conducted. For the great benefit of these parturients, medical therapy needs to be optimized in the peripartum period and RA should ideally be utilized with the use of vasoactive agents in a judicious manner. Operative delivery should ideally be reserved for obstetric indications with careful consideration of maternal and fetal wellbeing [21,22].

A previous study on congenital heart disease using the cardiac risk index scoring method observed that the estimated risk of cardiac events during pregnancy was 5% with a score of 0, 27% with a score of 1, and 75% with a score of more than 1 [5]. This cardiac risk index was used for parturients with either congenital or other forms of heart disease [5,11]. The findings from our study provide validation of the risk score for women with RHD, especially those delivering by CS. This simple scoring system will help anesthesiologists, especially from countries where RHD is still endemic to predict adverse cardiac events in the peripartum period. 

Our study has the inherent limitations of a retrospective study. Although data was collected carefully using standardized data collection forms, the completeness of data was dependent on the documentation available in the medical records. In addition, the applicability of our results is limited since this study was performed in a single tertiary centre. Nevertheless, our study is one of the only studies in the literature that focuses on anaesthesia management of parturients with valvular heart disease from a developing country where RHD is endemic.

In addition, our attempt to characterize the prevalence of maternal cardiac and neonatal complications in women with RHD revealed that previously proposed risk stratification for parturients with congenital heart disease can provide a similar predictive value in a setting where anaesthesia care is provided to patients with RHD.

## Conclusions

We observed that although maternal and neonatal mortality was low, there was considerable morbidity in parturients suffering from valvular heart disease and in their newborns. Multidisciplinary planning and elective CS preferably under RA appear to be a safe approach for high-risk parturients. Risk stratification in women with RHD will help in predicting peripartum complications. However, further prospective randomized controlled studies should be conducted to evaluate the impact of anaesthesia selection on maternal and neonatal complications in this patient population. 
